# Larval ecology and infestation indices of two major arbovirus vectors, *Aedes aegypti* and *Aedes albopictus* (Diptera: Culicidae), in Brazzaville, the capital city of the Republic of the Congo

**DOI:** 10.1186/s13071-020-04374-x

**Published:** 2020-09-25

**Authors:** Theodel A. Wilson-Bahun, Basile Kamgang, Arsène Lenga, Charles S. Wondji

**Affiliations:** 1 Centre for Research in Infectious Diseases (CRID), P.O. Box 13591, Yaoundé, Cameroon; 2 Laboratory of Biodiversity and Animal Ecology, Department of Animal Biology and Physiology, Faculty of Sciences and Technology, Marien Ngouabi University, P.O. Box 69, Brazzaville, Congo; 3 Liverpool School of Tropical Medicine, Pembroke Place, Liverpool, L3 5QA UK

**Keywords:** *Aedes aegypti*, *Aedes albopictus*, Larval ecology, Transmission risk, Republic of the Congo

## Abstract

**Background:**

Invasive mosquito species, such as *Aedes albopictus* in Congo can affect the distribution of native species, changing the vector composition and pattern of disease transmission. Here, we comparatively establish the geographical distribution and larval habitat preference of *Ae. aegypti* and *Ae. albopictus* and the risk of arbovirus disease outbreaks using *Stegomyia* indices in the city of Brazzaville, the capital of the Republic of the Congo.

**Methods:**

Human dwelling surveys of water-holding containers for immature stages of *Aedes* was carried out in December 2017 in Brazzaville through a random cluster sampling method. A total of 268 human dwellings distributed in 9 boroughs and 27 neighbourhoods were surveyed across the city.

**Results:**

Overall, 455 potential larval habitats were surveyed. Both *Ae. aegypti* and *Ae. albopictus* were collected across the city with an overall high prevalence of *Ae. aegypti* (53.1%) compared to *Ae. albopictus* (46.9%). Geographical distribution analysis showed that *Ae. aegypti* was more abundant (mean = 6.6 ± 1.4) in neighbourhoods located in downtown, while the abundance of *Ae. albopictus* was low (mean = 3.5 ± 0.6) in suburbs. Peridomestic containers, especially discarded tanks, were the most strongly colonized productive larval habitat for both mosquito species with the prevalence of 56.4% and 53.1% for *Ae. aegypti* and *Ae. albopictus*, respectively. Globally, the house index (HI), Breteau index (BI) and container index (CI) were high for *Ae. aegypti* (26.6%, 38.4% and 22.6%) and *Ae. albopictus* (33.3%, 49.6% and 26.6%) compared to the transmission risk threshold (5%, 5% and 20%) established by the WHO/PAHO. Overall, pupae-based indices (the pupae index and the pupae per person index) were not significantly different between *Ae. aegypti* (273.4% and 23.2%) and *Ae. albopictus* (228.8% and 19.5%).

**Conclusions:**

The findings of this study suggest a high risk for transmission of arbovirus diseases in Brazzaville and call for an urgent need to implement vector control strategies against these vectors in the Republic of the Congo.
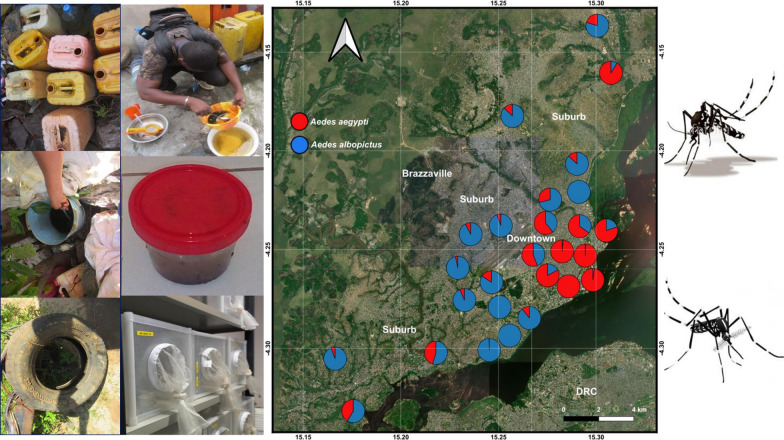

## Background

Mosquito-borne diseases such as dengue, chikungunya, yellow fever and Zika are increasing concerns in most tropical and sub-tropical regions. Dengue fever is the most important of these diseases worldwide. One last estimate indicates that, around 390 million dengue infections occur annually leading to 96 million (67–136 million) clinical cases [1]. In 2016, Zika virus was declared as a health emergency of international concern by WHO [2] because of the association of this virus with microcephaly [3], Guillain–Barré syndrome [4] and myelitis [5]. The burden of these arbovirus infections in Africa remains unknown except for yellow fever that a modelling study based on Africa data sources estimated in 2013, 84,000–170,000 severe cases, of which 29,000–60,000 were fatal [6]. Formerly, dengue, Zika, and chikungunya were considered as scarce in West-Central Africa despite of the presence of major vector *Aedes aegypti* Linnaeus. However, in the past decade, several outbreaks caused by these viruses have been reported in West-Central African countries [7–14] suggesting a possible change in the dynamics of these diseases. These viruses are transmitted to vertebrates including humans mainly by the bite of an infected mosquito belonging to the genus *Aedes* notably *Ae. aegypti* and *Aedes albopictus* (Skuse). *Aedes aegypti* is native from Africa forests [15], and is nowadays found in sylvan and human-dominated environments. Two subspecies of *Ae. aegypti* were formally identified by Mattingly [16]. The dark form, *Ae. aegypti formosus*, confined to the African forest and the light form, *Ae. aegypti aegypti*, found in human-dominated habitats primarily outside Africa [16]. The Sylvan population of *Ae. aegypti* breed in natural containers such as rock pools, tree holes and leaf axils [16–18] and rarely feed on humans [15], whereas domestic populations feed mainly on humans, mate and rest indoors, breed in man-made containers in and around human dwellings [19]. Generally, *Ae. aegypti* collected in Central Africa whatever the environment matches *Ae. aegypti formosus* [20, 21] suggesting that in Central Africa, two types of *Ae. aegypti formosus*, domestic and sylvan, co-occur. On the other hand, *Ae. albopictus*, originated from south east Asian forest, has invaded all the continents in past 30–40 decades [22]. This species was first reported in Central Africa in the early 2000s [23], and nowadays, is present in almost all countries of the region including the Republic of the Congo [24, 25]. *Aedes albopictus* is the dominant species of *Aedes* in most urban areas located under 6°N in Central Africa where it tends to replace the native species *Ae. aegypti* [25–28]. The coexistence of *Ae. aegypti* and *Ae. albopictus* sharing often the same larval habitats has been documented in Central Africa [26, 27, 29, 30]. Nevertheless, it was demonstrated that *Ae. aegypti* prefers man-made containers located in areas with high building density while *Ae. albopictus* rather prefers larval habitats surrounded by vegetation [27, 30, 31]. Interestingly, the emergence of dengue, Zika and chikungunya outbreaks in urban areas in Central Africa coincided with the invasion of the region by *Ae. albopictus* [32]. Indeed, *Ae. albopictus* was detected as the main vector during a concurrent dengue/chikungunya outbreak in Gabon in 2007 [9, 33]. Both *Ae. aegypti* and *Ae. albopictus* were found infected with chikungunya virus during the massive outbreak that occurred in Brazzaville in 2011 with 11,000 cases [13, 24]. Recently in 2019, *Ae. albopictus* was suspected as the main vector during the chikungunya outbreak affecting several locations in the Republic of the Congo [34]. It was also demonstrated that both *Ae. aegypti* and *Ae. albopictus* collected in Brazzaville are able to transmit yellow fever virus [35], Zika virus [36], and dengue virus [37]. As there is no efficient vaccine (apart from yellow fever) and specific treatment against these diseases, vector control remains the cornerstone to prevent outbreaks. However, implementing vector control strategies requires extensive background information on the larval ecology of *Aedes* species. However, in the Republic of the Congo, no such data are available apart from preliminary studies showing the co-occurrence of *Ae. aegypti* and *Ae. albopictus* across the country with a predominance of *Ae. albopictus* in all locations except in Brazzaville [25] and the dominance of *Ae. albopictus* in two periurban neighbourhoods from Brazzaville irrespective of the season [38]. Here, we present an extensive analysis of the levels of infestation, detailed comparative data of larval ecology, and geographical distribution of *Ae. aegypti* and *Ae. albopictus* in Brazzaville, the capital city of the Republic of the Congo to improve the control of these vectors and help prevent other arbovirus outbreaks in this major city.

## Methods

### Study area

**Fig. 1:**
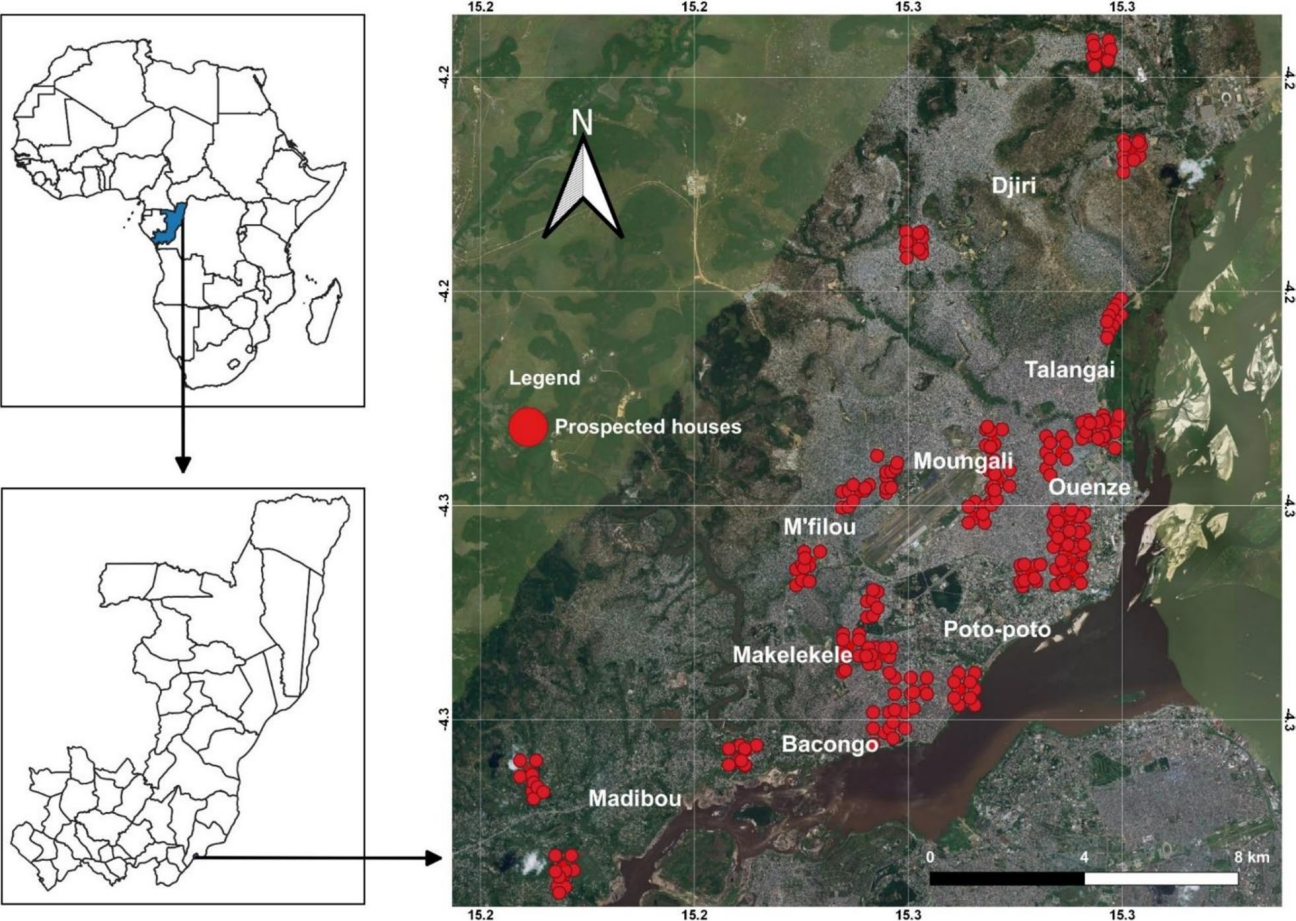
Map of Brazzaville indicating mosquito sampling sites

Surveys were carried out in Brazzaville (4°16′04′′S, 15°16′31′′E), the capital city of the Republic of the Congo (Fig. 1). The city is located along the Congo River and spans an area of 263.9 km^2^ with a population estimation of 1.4 million inhabitants. Brazzaville is laid out concentrically: the downtown is modern with urban buildings containing most administrative and commercial structures, while the suburb is unplanned and sparsely populated. Brazzaville is subdivided into 9 boroughs: Makélékélé; Bacongo; Poto-Poto; Moungali; Ouenzé; Talangaï; M’filou; Madibou; and Djiri. Each borough comprises several neighbourhoods. The climate is humid tropical with four seasons: a short dry season from January to February; a short rainy season from March to May; a long dry season from June to September; and a long rainy season from October to December. The mean annual precipitation ranges between 1500–1800 mm and the mean annual temperature is 25 °C [39].

### Sampling and entomological surveys

Entomological investigations were carried out in November/December 2017, corresponding to the long rainy season period in which *Aedes* mosquitoes are at maximum densities. Surveys were undertaken in clusters of dwellings sampled randomly. Each cluster consisted of 10 dwellings per neighbourhood randomly selected (by drawing lots). In each borough, 3 neighbourhoods were randomly selected by drawing lots, and boroughs were categorized, based on geographical position, the downtown (city centre with high building density) or suburb (periphery area of the city characterised by high vegetation density compare to downtown). The downtown is located in Poto-Poto, Moungali, and Ouenzé boroughs, while the suburb is located in Makélékélé, Bacongo, M’filou, Madibou, Talangaï and Djiri. Two rural neighbourhoods were surveyed in Madibou (Fig. 1, Table 1). During the surveys, each selected dwelling was geo-referenced with a global positioning system (GPS, Garmin Etrex 10), surveyed to record all natural and/or artificial containers with water (potential containers), and those containing at least one larvae or pupae (positive containers). Positive larval habitats were also geo-referenced. For each potential/positive container, the type of container, the volume of container (low, < 5 l; medium, 5–50 l; and high, > 50 l) the volume of water inside the container (low, < 5 l; medium, 5–50 l; and high, > 50 l), the source of the water inside the container (tap or rain) and the quality of water [clear; turbid (cloudy water), and polluted (foul smelling water with organic matter and/or with the presence of a layer of oil)] inside the container, the presence of plant debris inside the container, the presence of the vegetation (grass, tree or shrub) in the immediate vicinity of the container, the shade (full, partial or none), the colour (transparent, dark or light colour) of the container, the material (metal, plastic, rubber or miscellaneous), the distance between the container and the nearest building (0–5 m, 5–10 m), the distance of container to the nearest plant (grass, tree or shrub) (0–5 m, 5–10 m) and to the ground (0–1 m, 1–3 m) were noted as well the number of inhabitants per dwelling. On basis of the nature, the source, and the use of the water, potential containers were classified into three categories: domestic; peridomestic; and natural. Domestic containers (e.g. storage tanks) were defined as human-filled receptacles, while peridomestic (e.g. discarded tanks and used tyres), and natural receptacles (e.g. rock and tree holes, leaf axils) were those filled by rain [27]. Larvae and pupae found per container were collected, referenced and transported to the insectary and isolated from predators such as *Culex* (*Lutzia*) *tigripes.* Pupae per container were counted, isolated to larvae, and maintained until emergence to adults. Larvae were also reared to adults. Emerged adults per larval habitat were morphologically identified using a suitable taxonomic key [40, 41]. The proportion of larvae and pupae, or pupae of each *Aedes* species was estimated based on the number of specimens identified for each species divided by the total number of larvae and pupae, or pupae of *Aedes* spp. identified.

**Table 1:** Location of surveyed boroughs and neighbourhoods in Brazzaville

Borough	Neighbourhood	Geographical coordinates		Location
Bacongo	Mpissa	04°18′186″S	015°14′668″E	Suburb
	Saint Pierre Clavaire	04°17′614″S	015°14′998″E	Suburb
	Dahomey	04°17′429″S	015°15′854″E	Suburb
Makélékélé	Ngangouoni	04°16′890″S	015°14′222″E	Suburb
	Mokondzi Ngouaka	04°17′100″S	015°14′688″E	Suburb
	Diata	04°16′349″S	015°14′437″E	Suburb
Poto-Poto	Tsinguidi	04°15′893″S	015°16′581″E	Downtown
	Quartier 31	04°16′030″S	015°17′155″E	Downtown
	Quartier 32	04°15′773″S	015°17′309″E	Downtown
Moungali	10 maisons	04°14′499″S	015°16′115″E	Downtown
	Matsoua	04°14′783″S	015°16′147″E	Downtown
	Mounkondo	04°14′186″S	015°16′167″E	Downtown
Ouenzé	Quartier 51	04°15′182″S	015°17′335″E	Downtown
	Quartier 52	04°15′282″S	015°17′151″E	Downtown
	Bouemba	04°14′115″S	015°17′041″E	Downtown
Talangaï	Fleuve Congo	04°13′960″S	015°17′818″E	Suburb
	Quartier 64	04°13′912″S	015°17′645″E	Suburb
	Quartier 68	04°12′212″S	015°17′891″E	Suburb
M′filou	Moutabala	04°15′907″S	015°13′402″E	Suburb
	Indzuli	04°14′890″S	015°14′152″E	Suburb
	Ngambio	04°14′698″S	015°14′724″E	Suburb
Madibou	Kombé	04°20′226″S	015°10′098″E	Rural
	Kibina	04°18′668″S	015°09′665″E	Rural
	Mafouta	04°18′461″S	015°12′747″E	Suburb
Djiri	Manianga	04°09′983″S	015°18′111″E	Suburb
	Makabandilou	04°08′552″S	015°17′693″E	Suburb
	Matari	04°11′295″S	015°15′078″E	Suburb

### Entomological indices

The level of infestation was estimated using traditional *Stegomyia* indices including the Breteau index (BI, the number of positive containers per 100 surveyed houses), house index (HI, the percentage of houses infested), and container index (CI, percentage of positive containers). Estimated thresholds of HI, BI and CI references were established by the WHO for dengue and yellow fever transmission. Whenever HI > 35%, BI > 50, and CI > 20%, the city is considered as high risk of urban transmission of yellow fever virus, whereas HI < 4%, BI < 5 and CI < 3%, indicated that the city is considered as low risk of the disease transmission [42]. Similarly, low HI < 0.1%, medium HI 0.1–5% and high HI > 5% were established for dengue transmission [43]. Additional indices based on absence/presence and number of pupae were also used including pupae index (PI, number of pupae per 100 surveyed houses) and pupae per person index (PPI, number of pupae per 100 inhabitants). The productivity of pupae in each container type was also assessed as defined (the number of pupae in each container type divided by the total number of pupae in all container types) [44].

### Data analysis

All statistical analyses were performed using R version 3.1.5 [45] and R studio version 1.1.463, at α = 0.05 level of significance. Variables defined as categorical variables were expressed by percentages and confidence intervals, and numeric variables were expressed by means and standard deviations. The Shapiro–Wilk test for normality was used to assess the distribution of the data. Because data were not normally distributed, non-parametric statistical tests were used to compare variables; Chi-square test was used for percentages and Wilcoxon rank sum test for means. A binary logistic regression model was used to assess the relationship between larval habitat characteristics and presence of immature stages (larvae and pupae) or pupae only of each *Aedes* species (Additional file 1: Table S1). Odds ratios (OR) and their 95% confidence intervals (95% CI) were estimated. While a negative binomial regression model was used to assess the relationship between larval habitat characteristics (Additional file 1: Table S1) and number of immature stages and pupae only of *Aedes* spp. (based on estimates and their standard deviations). Both models were computed first with all factors related to the larval habitat and the environment, and then, each model was refined using a stepwise procedure based on Akaikeʼs information criterion (AIC) [46]. The GPS coordinates of each surveyed house, and each positive container of both species, were projected onto a map using the open-source software QGIS (version 3.4.1 Madeira) [47].

## Results

### Pre-imaginal infestation

In total, 268 dwellings were surveyed in 27 neighbourhoods across Brazzaville, with 3076 inhabitants (Table 2). Among the surveyed dwellings, 111 (41.4%) were found positive, harbouring at least one positive container of *Aedes* (larva and/or pupa) (Table 2). A total of 3787 specimens of immature stages of *Aedes* were collected, comprising of 1993 (52.6%) *Ae. aegypti*, 1760 (46.5%) *Ae. albopictus* and 34 (0.9%) *Ae. vittatus* Bigot, 1861. Nevertheless, several other species were also found in association with these *Aedes* species, notably *Anopheles gambiae* Giles, 1902 (*s.l*.) (6 specimens), *Culex tigripes* De Grandpré & De Charmoy, 1900 (107 specimens) and *Culex* spp. (216 specimens).

**Table 2:** Levels of infestation of *Aedes* spp. in different locations in Brazzaville

Location	Inhabitants	Dwellings		*Aedes* spp.	*Ae. aegypti*	*Ae. albopictus*	*Ae. vittatus*
	*n*	Surveyed	Positive	*n*	*n*	*n*	*n*
Bacongo	369	30	9	285	3	282	0
Mpissa	137	10	4	233	1	232	0
Saint Pierre Clavaire	129	10	4	34	0	34	0
Dahomey	103	10	1	18	2	16	0
Makélékélé	287	28	13	364	34	330	0
Ngangouoni	82	10	7	133	9	124	0
Mokondzi Ngouaka	118	10	2	72	0	72	0
Diata	87	8	4	159	25	134	0
Poto-Poto	400	30	18	563	532	31	0
Tsinguidi	130	10	5	146	121	25	0
Quartier 31	128	10	5	118	118	0	0
Quartier 32	142	10	8	299	293	6	0
Moungali	496	30	13	661	341	320	0
10_maisons	147	10	6	435	264	171	0
Matsoua	149	10	2	43	24	19	0
Mounkondo	200	10	5	183	53	130	0
Ouenzé	327	30	14	892	805	87	0
Quartier 51	107	10	6	416	333	83	0
Quartier 52	105	10	6	318	316	2	0
Bouemba	115	10	2	158	156	2	0
Talangaï	411	30	11	276	80	196	0
Fleuve Congo	161	10	5	166	22	144	0
Quartier 64	131	10	4	89	58	31	0
Quartier 68	119	10	2	21	0	21	0
Mfilou	299	30	12	210	10	200	0
Moutabala	104	10	4	118	4	114	0
Indzuli	101	10	5	39	3	36	0
Ngambio	94	10	3	53	3	50	0
Madibou	242	30	10	170	45	91	34
Kombé	82	10	4	92	38	54	0
Kibina	75	10	3	33	2	31	0
Mafouta	85	10	3	45	5	6	34
Djiri	305	30	11	366	143	223	0
Manianga	93	10	2	103	94	9	0
Makabandilou	88	10	7	201	41	160	0
Matari	124	10	2	62	8	54	0
Total	3136	268	111	3787	1993	1760	34

*Abbreviation*: *n*, number of inhabitants and *Aedes* species per neighbourhoods and boroughs

### Types and prevalence of water-holding containers

**Fig. 2:**
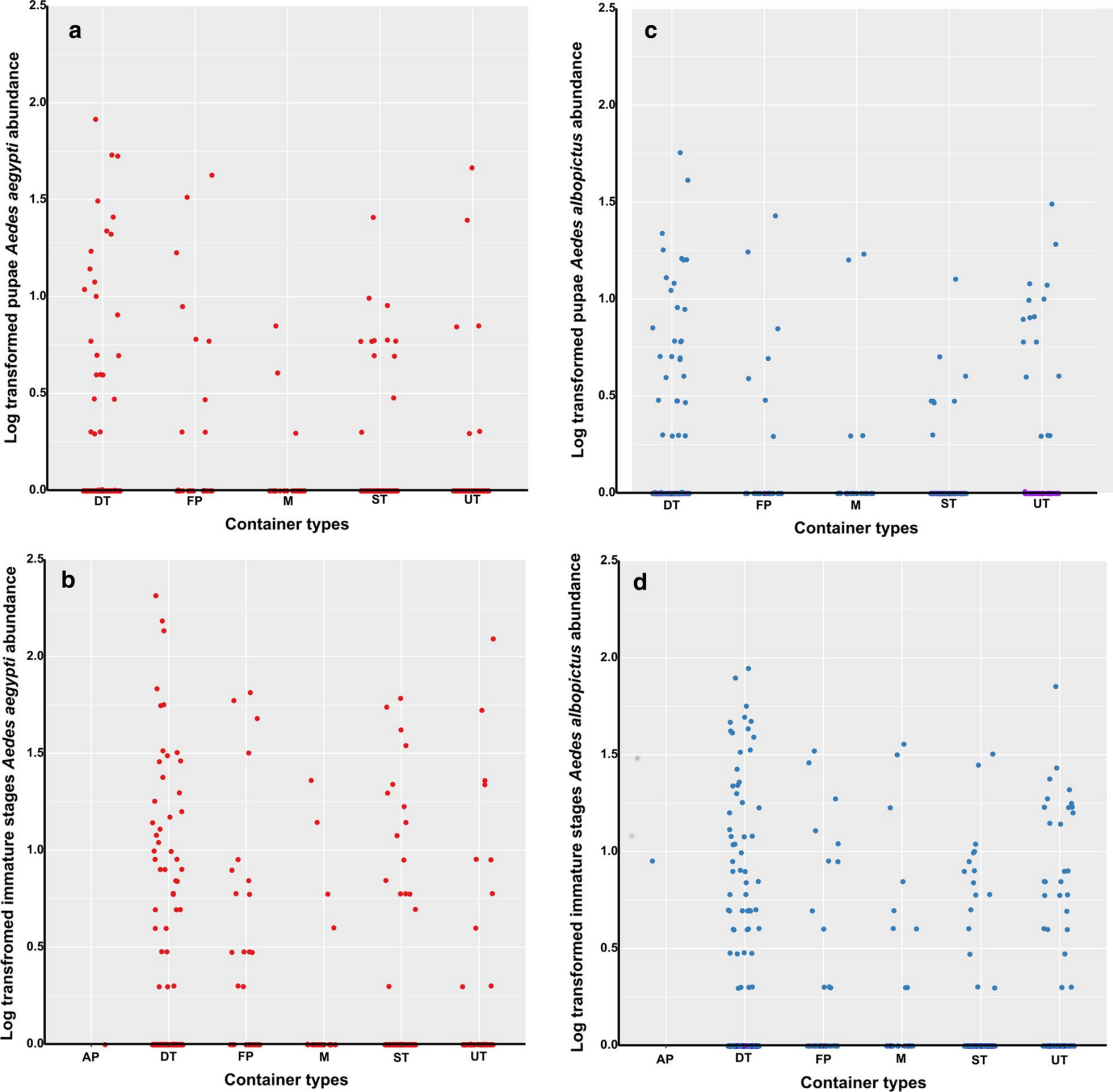
Total abundance of pupae and of immature stages of *Aedes aegypti* (**a**, **b**) and *Ae. albopictus* (**c**, **d**) per container type. *Abbreviations*: DT, discarded tanks; FP, flower-pots; M, miscellaneous; ST, storage tanks; and UT, used tires. Each dot represents the log_10_-transform of the abundance of containers infested by immature stages of *Ae. albopictus* and *Ae. aegypti*

A total of 455 potential breeding containers for *Aedes* spp. were surveyed, of which 176 (38.7%) were positive for immature stages of *Aedes* spp. (Table 3). Containers found during the surveys were grouped into three categories and five types (Table 3): domestic containers (water storage tanks, and flower-pots); peridomestic containers (discarded tanks, used tyres, and miscellaneous); and natural containers (axils of plants). Analysis revealed that *Ae. albopictus* (mean ± SD: 4.9 ± 1.2) was significantly more abundant than *Ae. aegypti* (mean ± SD: 3.6 ± 1.9) in used tyres (Wilcoxon rank sum test: *W* = 2199, *P* = 0.007) (Fig. 2). However, no significant difference was found in the prevalence of both species in the other container types (Wilcoxon rank sum test: *W* = 18094, *P* > 0.05) (Fig. 2). Discarded tanks were the most prevalent (Table 3), and the most productive water-holding container type for both *Ae. aegypti* (56.4% of pupae) and *Ae. albopictus* (53.1% of pupae) (Fig. 2). Nevertheless, no significant difference was observed in the pupae abundance of both species according to the container type (Wilcoxon rank sum test: *W* = 17414, *P* > 0.05) except for used tyres where *Ae. albopictus* pupae abundance (mean ± SD: 2.0 ± 0.6) was significantly higher (Wilcoxon rank sum test: *W* = 2050, *P* = 0.013) than those of *Ae. aegypti* (mean ± SD: 1.3 ± 0.7) (Fig. 2).

**Table 3:** Typology of containers and level of infestation of each container by *Ae. aegypti* and *Ae. albopictus* in Brazzaville

Location	Container types						Total *n* (%)
	Domestic		Peridomestic			Natural	
	Storage tanks *n* (%)	Flower-pots *n* (%)	Discarded tanks *n* (%)	Miscellaneous *n* (%)	Used tyres *n* (%)	Axils of plants *n* (%)	
Bacongo	7 (42.8)	1 (0)	17 (52.9)	3 (33.3)	5 (80.0)	0 (0)	33 (51.5)
Mpissa	4 (75.0)	0 (0)	8 (50.0)	1 (100)	2 (100)	0 (0)	15 (66.6)
Saint Pierre Clavaire	0 (0)	1 (0)	6 (50.0)	2 (0)	3 (66.6)	0 (0)	12 (41.6)
Dahomey	3 (0)	0 (0)	3 (66.6)	0 (0)	0 (0)	0 (0)	6 (33.3)
Makélékélé	9 (44.4)	6 (66.6)	14 (35.7)	11 (36.6)	11 (36.6)	0 (0)	51 (41.2)
Ngangouoni	9 (44.4)	2 (50.0)	5 (60.0)	3 (33.33)	7 (42.8)	0 (0)	26 (46.1)
Mokondzi Ngouaka	0 (0)	2 (50.0)	2 (0)	1 (100)	2 (0)	0 (0)	7 (28.6)
Diata	0 (0)	2 (100)	7 (28.6)	7 (28.6)	2 (50.0)	0 (0)	18 (38.8)
Poto-Poto	18 (27.8)	13 (61.5)	26 (42.3)	1 (0)	10 (30.0)	0 (0)	68 (39.7)
Tsinguidi	4 (0)	5 (60.0)	12 (25.0)	0 (0)	2 (0)	0 (0)	23 (26.1)
Quartier 31	0 (0)	5 (60.0)	7 (42.8)	1 (0)	3 (33.3)	0 (0)	16 (43.7)
Quartier 32	14 (35.7)	3 (66.6)	7 (71.4)	0 (0)	5 (40.0)	0 (0)	29 (48.3)
Moungali	15 (40.0)	2 (100)	15 (53.3)	1 (100)	9 (33.3)	0 (0)	42 (47.6)
10 maisons	9 (11.1)	2 (100)	5 (80.0)	1 (1)	3 (33.3)	0 (0)	20 (45.0)
Matsoua	3 (100)	0 (0)	5 (0)	0 (0)	1 (0)	0 (0)	9 (33.3)
Mounkondo	3 (66.6)	0 (0)	5 (80.0)	0 (0)	5 (40.0)	0 (0)	13 (61.5)
Ouenzé	18 (38.8)	5 (80.0)	12 (66.6)	1 (100)	3 (100)	0 (0)	39 (58.9)
Quartier 51	5 (80.0)	2 (100)	4 (75.0)	1 (100)	3 (100)	0 (0)	15 (86.6)
Quartier 52	8 (37.5)	1 (100)	5 (80.0)	0 (0)	0 (0)	0 (0)	14 (57.1)
Bouemba	5 (0)	2 (50.0)	3 (33.3)	0 (0)	0 (0)	0 (0)	10 (20.0)
Talangaï	11 (27.2)	0 (0)	21 (57.1)	1 (100)	4 (25.0)	0 (0)	37 (45.9)
Fleuve Congo	0 (0)	0 (0)	12 (50.0)	0 (0)	4 (25.0)	0 (0)	16 (43.7)
Quartier 64	4 (0)	0 (0)	8 (75.0)	1 (100)	0 (0)	0 (0)	13 (53.8)
Quartier 68	7 (42.8)	0 (0)	1 (0)	0 (0)	0 (0)	0 (0)	8 (37.5)
Mfilou	12 (0)	0 (0)	36 (22.2)	5 (80.0)	16 (50.0)	1 (100)	70 (30.0)
Moutabala	12 (0)	0 (0)	11 (36.4)	0 (0)	2 (100)	1 (100)	26 (26.9)
Indzuli	0 (0)	0 (0)	17 (5.88)	5 (80.0)	10 (40.0)	0 (0)	32 (28.1)
Ngambio	0 (0)	0 (0)	8 (37.5)	0 (0)	4 (50.0)	0 (0)	12 (41.6)
Madibou	9 (0)	6 (0)	33 (27.3)	2 (50.0)	8 (37.5)	0 (0)	58 (22.4)
Kombé	0 (0)	2 (0)	14 (28.6)	1 (100)	2 (50.0)	0 (0)	19 (31.6)
Kibina	0 (0)	0 (0)	11 (18.2)	0 (0)	5 (40.0)	0 (0)	16 (25.0)
Mafouta	9 (0)	4 (0)	8 (37.5)	1 (0)	1 (0)	0 (0)	23 (13.0)
Djiri	25 (8.0)	1 (100)	20 (55.0)	5 (0)	6 (50.0)	0 (0)	57 (29.8)
Manianga	2 (0)	0 (0)	6 (50.0)	1 (0)	0 (0)	0 (0)	9 (33.3)
Makabandilou	2 (50.0)	1 (100)	13 (53.8)	3 (0)	4 (50.0)	0 (0)	23 (20.9)
Matari	21 (4.8)	0 (0)	1 (100)	1(0)	2 (50.0)	0 (0)	25 (12.0)
Total	124 (24.2)	34 (55.9)	194 (41.7)	30 (43.3)	72 (40.3)	1 (100)	455 (38.7)

*Abbreviations*: *n*, number of potential containers prospected; %, denotes percentage of positive containers (infested by *Aedes* species)

### Risk factors for the presence and the abundance of *Aedes* species

Binary logistic regression analysis showed that several factors influence the presence of immature stages of *Ae. aegypti* and *Ae. albopictus* as well as the presence of pupae of both species (Table 4). Indeed, the turbid aspect of water (OR: 2.38, 95% CI: 1.29–4.41, *P* = 0.005), the location of neighbourhoods (suburb and rural) (OR: 0.12, 95% CI: 0.071–0.22, *P* < 0.0001 and OR: 0.11, 95% CI: 0.03–0.30, *P* < 0.0001, respectively), the presence of surrounding vegetation (OR: 2.89, 95% CI: 1.59–5.44, *P* = 0.0007), and the container colour (transparent) (OR: 0.25, 95% CI: 0.07–0.94, *P* = 0.03) were significantly associated with the presence of immature stages of *Ae. aegypti* whereas only the turbid aspect of water (OR: 2.06, 95% CI: 1.20–3.53, *P* = 0.008), and the presence of surrounding vegetation (OR: 2.97, 95% CI: 1.77–5.08, *P* < 0.0001) was significantly associated with the presence of immature stages of *Ae. albopictus* (Table 4). The presence of pupae of *Ae. aegypti* was significantly associated with the turbid aspect of water (OR: 2.54, 95% CI: 1.24–5.19, *P* = 0.01), the location of neighbourhoods (suburb and rural) (OR: 0.07, 95% CI: 0.03–0.15, *P* < 0.0001 and OR: 0.08, 95% CI: 0.02–0.27, *P* = 0.0002, respectively), the presence of surrounding vegetation (OR: 5.81, 95% CI: 2.75–13.14, *P* < 0.0001), the container colour (light and transparent) (OR: 0.25, 95% CI: 0.08–0.85, *P* = 0.02 and OR: 0.14, 95% CI: 0.03–0.60, *P* = 0.007, respectively) the source of water inside de container (tap) (OR: 0.38, 95% CI: 0.15–0.89, *P* = 0.03) and the material of the container (OR: 0.08, 95% CI: 0.02–0.38, *P* = 0.001) whereas that of *Ae. albopictus* was significantly associated to the turbid aspect of water (OR: 2.28, 95% CI: 1.23–4.16, *P* = 0.03), and the location of neighbourhood (suburb) (OR: 1.93, 95% CI: 1.03–3.74, *P* = 0.04) (Table 5).

**Table 4:** Factors influencing the presence of larvae and/or pupae of *Aedes aegypti* and *Aedes albopictus* in the larval habitat

Factor	Modality	*Ae. aegypti*		*Ae. albopictus*	
		OR	95% CI	OR	95% CI
Neighbourhood location	Downtown	Ref	Ref	Ref	Ref
	Suburb	0.12***	0.071–0.22	1.37	0.84–0.29
	Rural	0.11***	0.03–0.30	0.52	0.19–1.29
Container types	Axils of plants	Ref	Ref	Ref	Ref
	Discarded tanks	3.75 × 10^6^	1.73 × 10^−206^–na	5.05 × 10^−8^	na–1.15 × 10^205^
	Flower-pots	5.32 × 10^6^	3.55 × 10^−206^–na	4.85 × 10^−8^	na–8.87 × 10^204^
	Miscellaneous	2.21 × 10^6^	1.66 × 10^−206^–na	3.20 × 10^−8^	na–5.65 × 10^204^
	Storage tanks	1.48 × 10^6^	7.58 × 10^−207^–na	1.09 × 10^−8^	na–1.83 × 10^204^
	Used tyres	8.77 × 10^5^	8.72 × 10^−207^–na	5.59 × 10^−8^	na–1.14 × 10^205^
Container colour	Dark	Ref	Ref	na	na
	Light	0.62	0.21–1.94	na	na
	Transparent	0.25*	0.07–0.94	na	na
Water quality	Clear	Ref	Ref	Ref	Ref
	Turbid	2.38**	1.29–4.41	2.06**	1.20–3.53
	Polluted	1.69 × 10^−7^	na–9.71 × 10^20^	1.99 × 10^−7^	na–2.03 × 10^23^
Water source	Tap	na	na	2.49	1.01–6.74
	Rain	na	na	Ref	Ref
Presence of surrounding vegetation	Yes	2.89***	1.59–5.44	2.97***	1.77–5.08
	No	Ref	Ref	Ref	Ref
Distance to nearest building	0–5 m	na	na	Ref	Ref
	5–10 m	na	na	0.57	0.29–1.08

*P < 0.05, **P < 0.01, ***P < 0.0001

*Abbreviations*: na, not applicable; OR, odd ratio; CI, 95% confidence interval; Ref, reference (the reference for each factor were randomly selected by the software) is a comparator group, distances in meters (m)

**Table 5:** Factors influencing the presence of pupae of *Aedes aegypti* and *Aedes albopictus* in the larval habitat

Factor	Modality	*Ae. aegypti*		*Ae. albopictus*	
		OR	95% CI	OR	95% CI
Neighbourhoods location	Downtown	Ref	Ref	Ref	Ref
	Suburb	0.07***	0.03–0.15	1.93*	1.03–3.74
	Rural	0.08***	0.02–0.27	1.03	0.33–2.95
Container types	Axils of plants	na	na	Ref	Ref
	Discarded tanks	na	na	6.54 × 10^−9^	na–6.34 × 10^203^
	Flower-pots	na	na	6.79 × 10^−9^	na–4.75 × 10^203^
	Miscellaneous	na	na	5.19 × 10^−9^	na–4.59 × 10^203^
	Storage tanks	na	na	2.01 × 10^−9^	na–1.47 × 10^203^
	Used tyres	na	na	5.97 × 10^−9^	na–4.18 × 10^203^
Container colour	Dark	Ref	Ref	na	na
	Light	0.25*	0.08–0.85	na	na
	Transparent	0.14**	0.03–0.60	na	na
Container material	Metal	Ref	Ref	Ref	Ref
	Plastic	1.84	0.76–4.74	1.01	0.52–2.00
	Rubber	0.08**	0.02–0.38	na	na
	Miscellaneous	1.35	0.29–5.52	0.25	0.03–1.02
Water quality	Clear	Ref	Ref	Ref	Ref
	Turbid	2.54*	1.24–5.19	2.28*	1.23–4.16
	Polluted	3.95 × 10^−7^	na–2.82 × 10^21^	4.07 × 10^−7^	na–8.41 × 10^23^
Water source	Tap	0.38*	0.15–0.89	na	na
	Rain	Ref	Ref	na	na
Presence of surrounding vegetation	Yes	5.81***	2.75–13.14	1.98	1.08–3.77
	No	Ref	Ref	Ref	Ref
Distance to nearest building	0–5 m	na	na	Ref	Ref
	5–10 m	na	na	0.5	0.21–1.08

* *P* < 0.05, ** *P* < 0.01, *** *P* < 0.0001

*Abbreviations*: na, not applicable; OR, odd ratio; CI, confidence interval; Ref, reference (the reference for each factor were randomly selected by the software) is a comparator group; distances in meters (m)

Furthermore, negative binomial regression was used to explore the factors which can influence the number of pupae of *Ae. aegypti* and *Ae. albopictus* inside the containers. Analysis revealed that the number of pupae of *Ae. aegypti* was positively influenced by the source of water (tap) (estimate = 4.06, *P* = 0.0003), the presence of plant residues inside the container (estimate = 9.33, *P* < 0.0001), the presence of surrounding vegetation (estimate = 15.56, *P* < 0.0001), the volume of water inside the container (estimate = 4.79, *P* < 0.0001), the container volume (estimate = 23.68, *P* < 0.0001), the distance to the nearest plant (0–5 m and 5–10 m) (estimate = 19.56, *P* < 0.0001 and estimate = 18.94, *P* < 0.0001, respectively), the distance to the nearest building (5–10 meters) (estimate = 5.69, *P* < 0.0001), the distance to ground (1–3 m) (estimate = 4.94, *P* = 0.03), and the neighbourhood location (rural) (estimate = 3.55, *P* = 0.003). The number of pupae of *Ae. albopictus* was also positively influenced by the turbid aspect of water (estimate = 1.98, *P* < 0.0001), the presence of surrounding vegetation (estimate = 1.47, *P* = 0.0004), and the neighbourhood location (suburb) (estimate = 1.38, *P* = 0.001) (Table 6). In contrast, the container colour (light and transparent) (estimate = − 11.27, *P* < 0.0001 and estimate = − 38.44, *P* < 0.0001, respectively), the absence of shade (estimate = − 9.75, *P* < 0.0001), but also the volume of water inside the container (estimate = − 84.72, *P* < 0.0001), the container volume (estimate = − 13.92, *P* < 0.0001) and the neighbourhood location (suburb) (estimate = − 25.81, *P* < 0.0001) were inversely associated to the number of *Ae. aegypti* pupae. While abundance of *Ae. albopictus* pupae was negatively associated to the absence of shade (estimate = − 1.12, *P* = 0.005) and the distance to nearest building (estimate = − 1.69, *P* = 0.001) (Table 6).

**Table 6:** Factors influencing the number of pupae of *Aedes aegypti* and *Aedes albopictus* in Brazzaville

Factor	Modality	*Ae. aegypti*			*Ae. albopictus*		
		Estimate	SE	*z*-value	Estimate	SE	*z*-value
Neighbourhoods location	Downtown	Ref	Ref	Ref	Ref	Ref	Ref
	Suburb	− 25.81***	0.7024	− 36.74	1.38**	0.43	3.25
	Rural	3.55**	1.19	2.970	0.52	0.73	0.72
Container types	Axils of plants	Ref	Ref	Ref	Ref	Ref	Ref
	Discarded tanks	170.22	6.71 × 10^7^	0	0.92	3.41	0.27
	Flower-pots	169.99	6.71 × 10^7^	0	1.42	3.47	0.41
	Miscellaneous	132.82	6.71 × 10^7^	0	− 0.95	3.47	− 0.28
	Storage tanks	148.40	6.71 × 10^7^	0	− 1.04	3.43	− 0.30
	Used tyres	175.28	6.71 × 10^7^	0	1.61	3.54	0.45
Container colour	Dark	Ref	Ref	Ref	Ref	Ref	Ref
	Light	− 11.27***	1.37	− 8.22	1.35	0.88	1.53
	Transparent	− 38.44***	1.58	− 24.28	− 0.14	0.99	− 0.14
Container material	Metal	Ref	Ref	Ref	na	na	na
	Plastic	15.01	0.89	16.83	na	na	na
	Rubber	na	na	na	na	na	na
	Miscellaneous	30.05	1.40	21.46	na	na	na
Container volume	Low (< 5 l)	Ref	Ref	Ref	na	na	na
	Medium (5–50 l)	− 13.92***	0.66	− 20.94	na	na	na
	High (> 50 l)	23.68***	1.58	14.91	na	na	na
Water volume	Low (< 5 l)	Ref	Ref	Ref	na	na	na
	Medium (5–50 l)	4.79***	0.80	5.96	na	na	na
	High (> 50 l)	− 84.72***	4.69	− 18.05	na	na	na
Water source	Tap	4.06***	1.13	3.58	na	na	na
	Rain	Ref	Ref	Ref	na	na	na
Water quality	Clear	Ref	Ref	Ref	Ref	Ref	Ref
	Turbid	− 0.97	0.73	− 1.325	1.98***	0.44	4.37
	Polluted	3.24	2.19	1.48	− 30.34	7.66×10^7^	0
Shade	None	− 9.75***	0.65	− 14.99	− 1.12**	0.39	− 2.82
	Partial	− 10.26	1.06	− 9.65	− 0.7451	0.59	− 1.25
	Full	Ref	Ref	Ref	Ref	Ref	Ref
Plant residues	Yes	9.33***	0.71	13.10	na	na	na
	No	Ref	Ref	Ref	na	na	na
Presence of surrounding vegetation	Yes	15.56***	0.96	16.14	1.47***	0.4212	3.49
	No	Ref	Ref	Ref	Ref	Ref	Ref
Distance to nearest plant	0–5 m	19.56***	1.04	18.71	na	na	na
	5–10 m	18.94***	2.09	9.02	na	na	na
	> 10 m	Ref	Ref	Ref	na	na	na
Distance to nearest building	0–5 m	Ref	Ref	Ref	Ref	Ref	Ref
	5–10 m	5.69***	0.83	6.77	− 1.69**	0.57	− 3.23
Distance to ground	0–1 m	Ref	Ref	Ref	na	na	na
	1–3 m	4.94*	2.38	2.07	na	na	na

* *P* < 0.05, ** *P* < 0.01, *** *P* < 0.0001

*Abbreviations*: na, not applicable; CI, confidence interval; Ref, reference (the reference for each factor were randomly selected by the software) is a comparator group; SE, standard error; distance in meters (m), volume in litres (l)

### Spatial distribution of *Aedes* species in Brazzaville

**Fig. 3:**
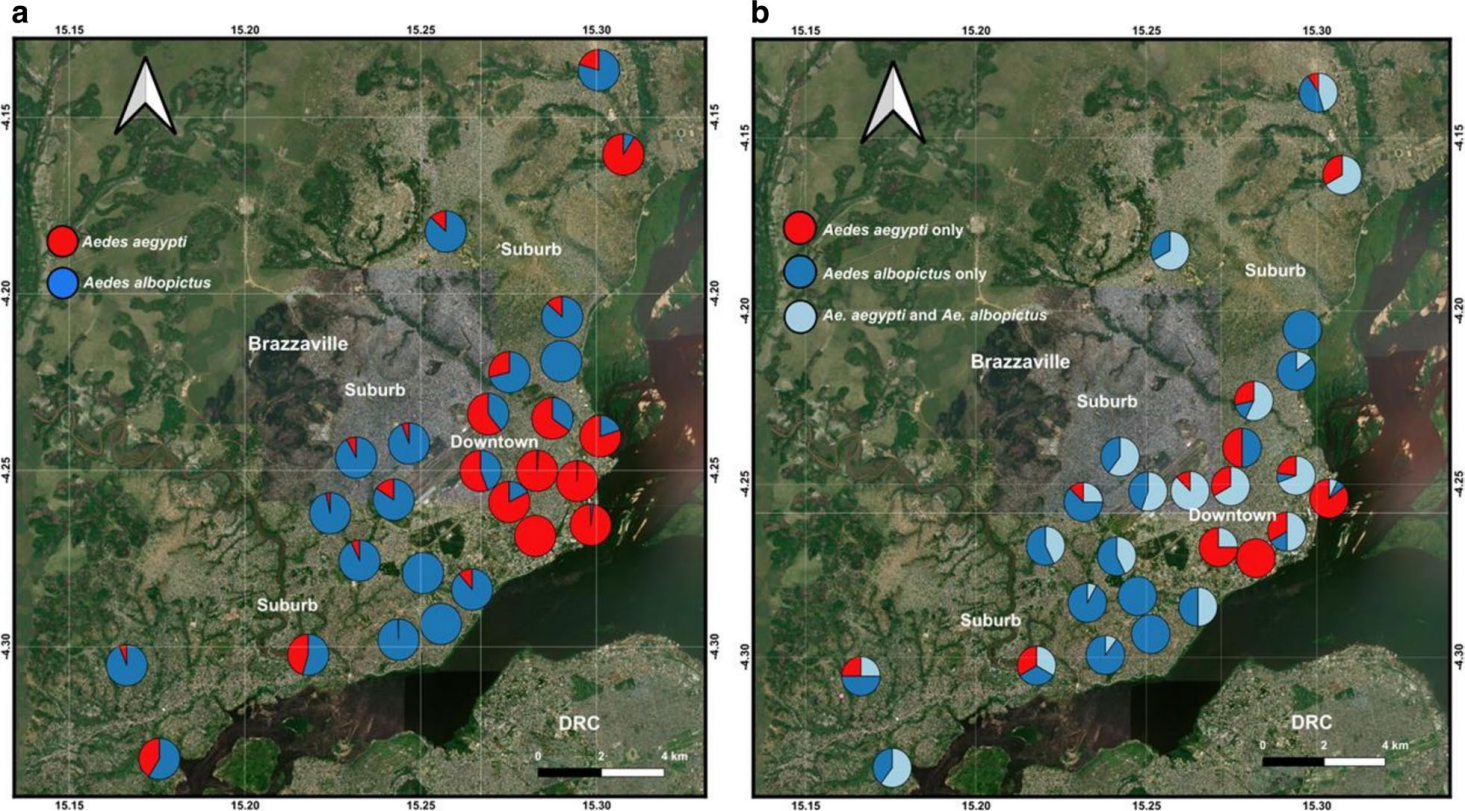
Geographical distribution of *Ae. aegypti* and *Ae. albopictus* in Brazzaville boroughs in 2017. **a** Spatial distribution and abundance of *Ae. aegypti* and *Ae. albopictus* according to the neighbourhoods. **b** Spatial distribution of positive larval habitats, and their occupancy (*Ae*. *aegypti* only; *Ae. albopictus* only; both *Ae. aegypti*; and *Ae. albopictus*) according to the neighbourhoods

Both *Ae. aegypti* and *Ae. albopictus* were found across the city (Fig. 3a) while *Ae. vittatus* was collected only in one periurban borough (Madibou) (Table 2). Overall, *Ae. aegypti* (52.6%) was significantly more prevalent (Wilcoxon rank sum test: *W* = 137840, *P* = 0.02) than *Ae. albopictus* (46.5%) throughout the city. However, when analysis was performed according to the borough, *Ae. albopictus* was significantly (Wilcoxon rank sum test: *W* = 177030, *P* < 0.0001) more abundant than *Ae. aegypti* in three boroughs (Bacongo, Makélékélé and M’filou), all located in the suburb of the city (Table 7). *Aedes aegypti*, was significantly (Wilcoxon rank sum test: *W* = 177030, *P* < 0.0001) more abundant than *Ae. albopictus* only in two boroughs located in downtown, Poto-Poto (*Ae. aegypti*, 7.8 ± 2.0; *Ae. albopictus*, 0.5 ± 0.2) and Ouenzé (*Ae. aegypti*, 20.6 ± 7.1; *Ae. albopictus*, 2.2 ± 0.8) (Fig. 3a, b; Table 7). On the other hand, no significant difference was detected for the prevalence of both *Ae. aegypti* and *Ae. albopictus* species respectively in four other boroughs: Moungali (located in downtown); Djiri; Madibou; and Talangaï (Wilcoxon rank sum test: *W* = 1505, *P* > 0.05) (Table 7). When an analysis was performed taking into account the environment, *Ae. aegytpi* (mean ± SD: 6.60 ± 1.38) was significantly more abundant than *Ae. albopictus* (mean ± SD: 3.00 ± 0.53) in downtown (Wilcoxon rank sum test: *W* = 13334, *P* = 0.0003) compared to suburb where *Ae. albopictus*, (mean ± SD: 3.5 ± 0.6) was significantly more abundant than *Ae. aegypti* (mean ± SD: 0.9 ± 0.3) (Wilcoxon rank sum test: *W* = 37334, *P* < 0.0001) (Fig. 3a, b; Table 7).

**Table 7:** Abundance of *Aedes aegypti* and *Aedes albopictus* species according to the borough

Location	Borough	*Ae. aegypti*		*Ae. albopictus*		*P-*value
		*n*	Mean ± SE	*n*	Mean ± SE	
Downtown	Moungali	341	8.12 ± 3.64	320	7.62 ± 2.15	0.513
	Ouenzé	805	20.64 ± 7.12	87	2.23 ± 0.83	< *0.001*
	Poto-Poto	532	7.82 ± 2.01	31	0.46 ± 0.22	< *0.001*
Suburb	Bacongo	3	0.09 ± 0.07	282	8.54 ± 3.42	< *0.001*
	Djiri	143	2.51 ± 1.16	223	3.91 ± 1.39	0.359
	Madibou	45	0.78 ± 0.45	91	1.57 ± 0.56	0.3717
	Makélékélé	34	0.67 ± 0.35	330	6.47 ± 1.55	< *0.001*
	M’filou	10	0.14 ± 0.05	200	2.86 ± 0.89	< *0.001*
	Talangaï	80	2.16 ± 1.07	196	5.29 ± 2.39	0.058

*Abbreviations*: *n*, number; SE, standard error; *P*-value, level of significance between mean numbers of *Ae. aegypti* and *Ae. albopictus* according to the boroughs. Statistically significant *P*-values are indicated in italic

### Infestation indices

**Fig. 4:**
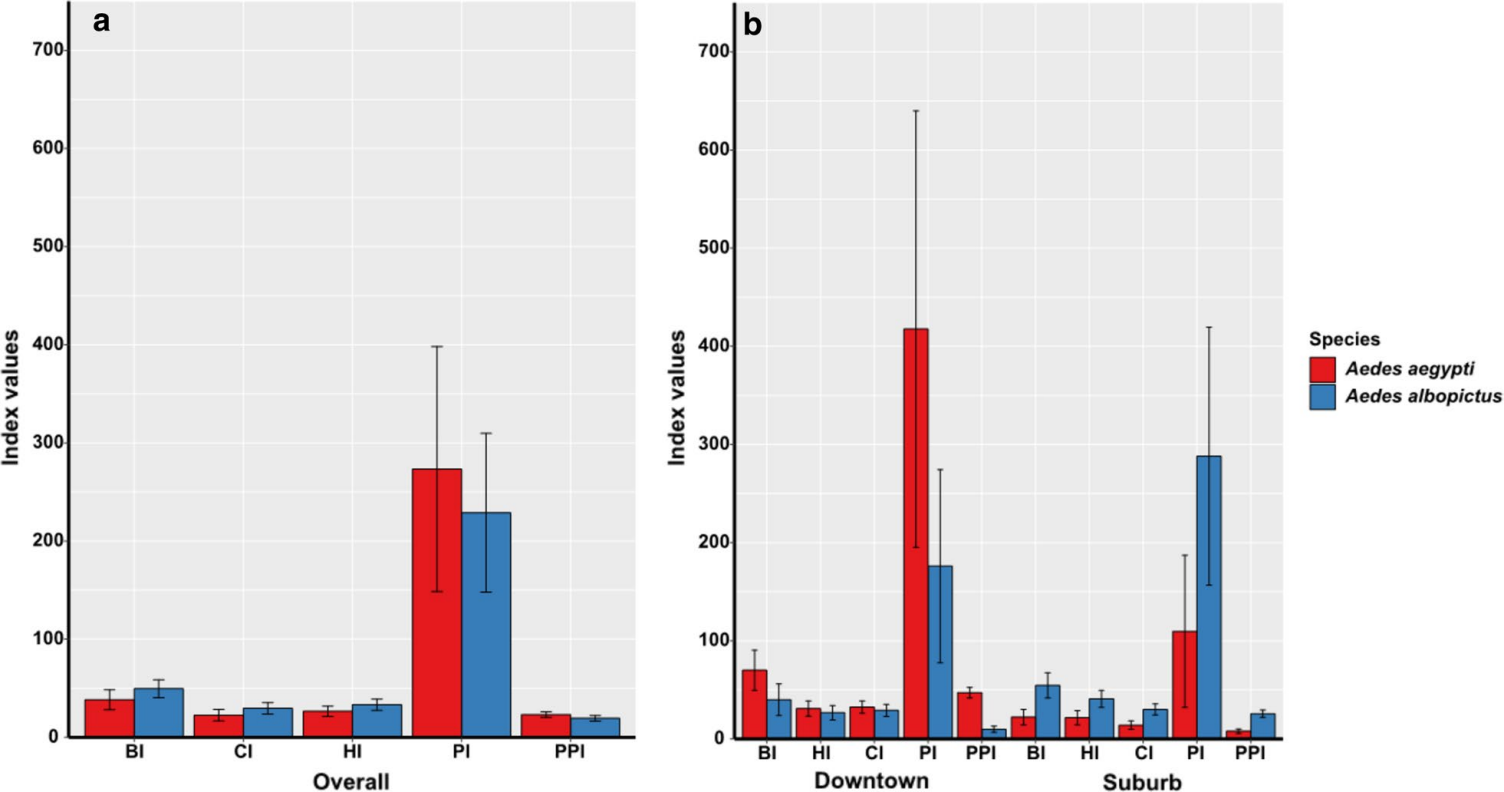
Level of infestation of *Ae. aegypti* and *Ae. albopictus* in Brazzaville in 2017. **a** Overall *Stegomyia* and pupae indices of *Ae. aegypti* and *Ae. albopictus* in Brazzaville. **b** Infestation indices of *Ae. aegypti* and *Ae. albopictus* according to the location of the borough. *Abbreviations*: BI, Breteau index (and 95% confidence interval); CI, container index (and 95% confidence interval); HI, house index (and 95% confidence interval); PI, pupae index (and 95% confidence interval); PPI, pupae per person index (and 95% confidence interval)

**Fig. 5:**
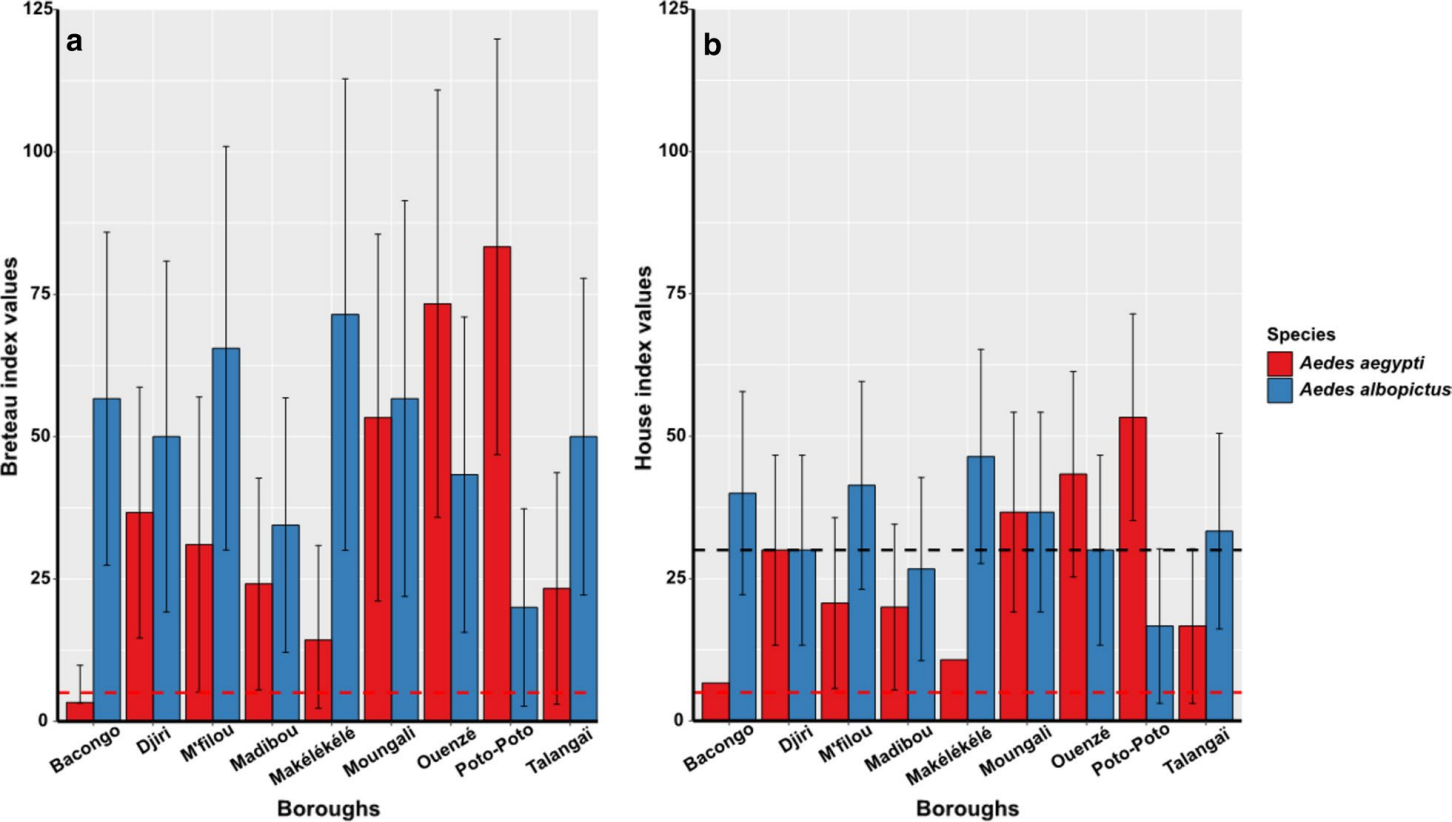
Infestation indices of *Aedes* spp. according to each borough. **a** BI, Breteau index (and 95% confidence interval). **b** HI, house index (and 95% confidence interval); black and red dashed line, represent the yellow fever and dengue epidemic threshold, respectively, defined by the WHO and PAHO

**Fig. 6:**
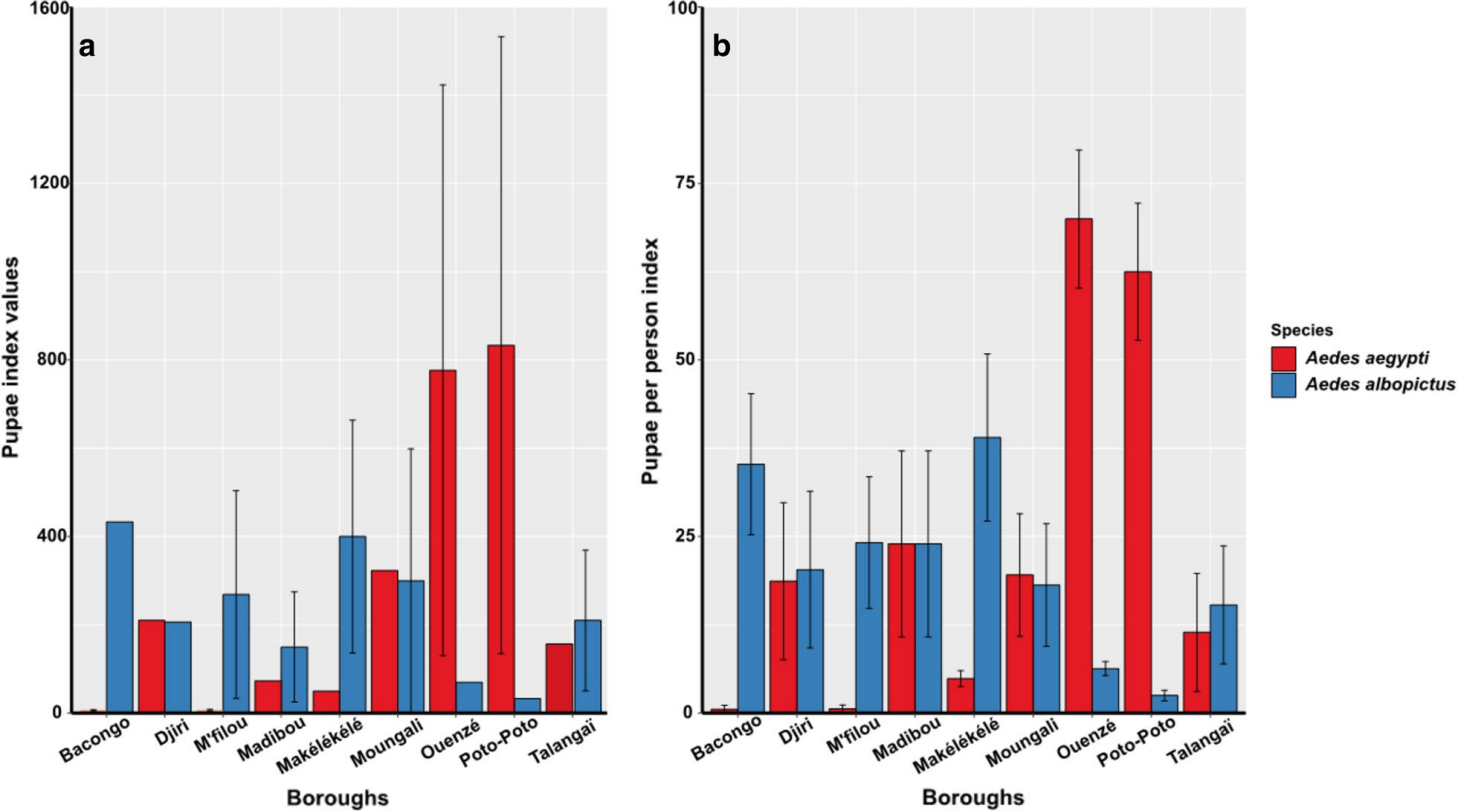
Pupae based indices of *Ae. aegypti* and *Ae. albopictus* according to each borough. **a** PI, pupae index (and 95% confidence interval). **b** PPI, pupae per person index (and 95% confidence interval)

*Stegomyia* and pupae indices are presented in Figs. 4, 5 and 6. Overall, the BI for *Ae. aegypti* and *Ae. albopictus* was 38.4% (95% CI: 28.3–48.4%) and 49.6% (95% CI: 40.5–58.7%) respectively, although the difference was not significant (Wilcoxon rank sum test: *W* = 3, *P* > 0.05) (Fig. 4a). Similarly, no significant difference (Wilcoxon rank sum test: *W* = 2, *P* > 0.05) was observed for BI values of *Ae. aegypti* and *Ae. albopictus* respectively in downtown (70%, 95% CI: 49.6–90.4%; 40%, 95% CI: 24–56%) and in the suburb (22.2%, 95% CI: 14.2–30.1%; 54.5%, 95% CI: 41.7–67.4%) (Fig. 4b). However, comparison of indices between both species according to the boroughs showed BI values for *Ae. albopictus* significantly higher compared to those for *Ae. aegypti* (Wilcoxon rank sum test: *W* = 2, *P* = 0.001) in six boroughs: Bacongo, Makélékélé, M’filou, Madibou, Talangaï and Djiri (Fig. 5a, Table 8) while BI values for *Ae. aegypti* were significantly higher compared to those for *Ae. albopictus* in Ouenzé and Poto-Poto (Wilcoxon rank sum test: *W* = 2, *P* = 0.001). However, no significant difference was found in BI values of both species apart in Moungali (Wilcoxon rank sum test: *W* = 0, *P* > 0.05) (Fig. 5a, Table 8). Globally, the CI was 22.6% (95% CI: 16.7–28.5%) and 29.6% (95% CI: 23.7–35.5%) for *Ae. aegypti* and *Ae. albopictus*, respectively, with no significant difference (Chi-square test: *χ*^2^ = 1.23, *df* = 2, *P* > 0.05) (Fig. 4a). However, the comparison of CI between both species according to the location of the boroughs (downtown *vs* suburb), showed that the CI was significantly higher in *Ae. aegypti* 32.4% (95% CI: 26.1–38.7%) than that of *Ae. albopictus* 29.1% (95% CI: 23.0–35.2%) in downtown (Chi-square test: *χ*^2^ = 11.707, *df* = 1, *P* = 0.0006). While in the suburb, the CI for *Ae. albopictus* 30% (95% CI: 24.3–35.7%) was significantly higher (Chi-square test: *χ*^2^ = 11.707, *df* = 1, *P* = 0.0006) than for *Ae. aegypti* 14% (95% CI: 9.7–18.3%) (Fig. 4b). The overall HI was 26.6% (95% CI: 21.3–31.9%) for *Ae. aegypti* and 33.3% (95% CI: 27.6–39.0%) for *Ae. albopictus* respectively with no significant difference (Chi-square test: *χ*^2^ = 1.23, *df* = 2, *P* > 0.05) (Fig. 4a). In contrast, HI values were significantly higher (Chi-square test: *χ*^2^ = 7.493, *df* = 1, *P* = 0.006) for *Ae. aegypti* 31% (95% CI: 23.3–38.6%) than for *Ae. albopictus* 26.8% (95% CI: 19.4–34%) in downtown areas, whereas the opposite situation was observed between *Ae. aegypti* 21.6% (95% CI: 14.3–28.8%) and *Ae. albopictus* 40.8% (95% CI: 32.1–49.4%) in suburb areas (Chi-square test: *χ*^2^ = 7.493, *df* = 1, *P* = 0.006) (Fig. 4b). The comparison of HI between both species according to the borough revealed that HIs were significantly higher (Chi-square test: *χ*^2^ = 46.713, *df* = 6, *P* < 0.0001) for *Ae. albopictus* than for *Ae. aegypti*, in six boroughs (Bacongo, Makélékélé, Madibou, M’filou and Talangaï) whereas the reverse situation was observed in Ouenzé and Poto-Poto (Chi-square test: *χ*^2^ = 46.713, *df* = 6, *P* < 0.0001) and no significant difference was found between two species in Moungali and Djiri (Chi-square test: *χ*^2^ = 0, *df* = 1, *P* > 0.05) (Fig. 5b, Table 8).

**Table 8:** Infestation indices of *Aedes* species according to the boroughs

Borough	*Ae. aegypti*				*Ae. albopictus*			
	BI (95% CI)	HI (95% CI)	PI (95% CI)	PPI (95% CI)	BI (95% CI)	HI (95% CI)	PI (95% CI)	PPI (95% CI)
Bacongo	3.33 (3.19–9.86)	6.67 (2.41–15.74)	6.67 (5.35–8)	0.54 (0–1.08)	56.67 (27.43–85.91)	40 (22.17–57.83)	433.33 (29.38–896.04)	35.23 (25.5–45.21)
Djiri	36.67 (14.66–58.68)	30 (13.32–46.68)	210 (5.8–425.8)	18.69 (7.59–29.79)	50 (19.18–80.81)	30 (13.32–46.68)	206.67 (14.11–427.44)	20.33 (9.23–31.43)
M’filou	31.03 (5.10–56.95)	20.69 (5.68–35.69)	6.9 (5.52–8.27)	0.62 (0.09–1.15)	65.52 (30.08–100.96)	41.38 (23.14–59.62)	268.96 (33.87–504.05)	24.15 (14.83–33.47)
Madibou	24.14 (5.54–42.74)	20 (5.44–34.56)	73.33 (26.97–173.64)	23.97 (10.81–37.13)	34.48 (12.14–56.82)	26.67 (10.57–42.76)	150 (25.06–274.93)	23.97 (10.81–37.13)
Makélékélé	14.29 (2.31–30.89)	10.71 (0.95–22.38)	50 (18.7–118.7)	4.88 (3.76–5.99)	71.43 (30.04–112.82)	46.43 (27.62–65.24)	400 (136.34–663.65)	39.02 (27.20–50.85)
Moungali	53.33 (21.14–85.52)	36.67 (19.13–54.20)	323.33 (66.82–713.49)	19.57 (10.89–28.23)	56.67 (21.91–91.43)	36.67 (19.13–54.20)	300 (1.49–598.51)	18.14 (9.48–26.81)
Ouenzé	73.33 (35.82–110.84)	43.33 (25.30–61.37)	776.67 (130.31–1423.02)	69.97 (60.18–79.76)	43.33 (15.64–71.02)	30 (13.32–46.68)	70 (22.15–162.15)	6.31 (5.33–7.29)
Poto–Poto	83.33 (46.83–119.83)	53.33 (35.17–71.49)	833.33 (134.67–1532)	62.5 (52.79–72.21)	20 (2.67–37.33)	16.67 (3.10–30.23)	33.33 (7.99–74.65)	2.5 (1.75–3.25)
Talangaï	23.33 (3–43.67)	16.67 (3.10–30.23)	156.67 (58.59–371.92)	11.43 (3.09–19.79)	50 (22.2–77.8)	33.33 (16.17–50.49)	210 (50.70–369.30)	15.33 (6.98–23.68)

*Abbreviations*: BI, Breteau index; HI, house index; PI, pupae index; PPI, pupae per person index; CI, confidence interval

The overall PI values were not significantly different (Chi-square test: *χ*^2^ = 1.23, *df* = 2, *P* > 0.05) between *Ae. aegytpi* 273.4% (95% CI: 148.5–398.3%) and *Ae. albopictus* 228.8% (95% CI: 147.9–309.7%) (Fig. 4a). Also, no significant difference (Chi-square test: *χ*^2^ = 1.12, *df* = 1, *P* > 0.05) was observed between PI values of *Ae. aegypti* and *Ae. albopictus* in both downtown (417%, 95% CI: 195.2–640%; 176%, 95% CI: 77.5–274.5%) and the suburb (109.6%, 95% CI: 32–187.1%; 288%, 95% CI: 156.6–419.4%) locations, respectively (Fig. 4b). However, detailed analysis according to the borough showed that, PI values were significantly higher for *Ae. albopictus* than for *Ae. aegypti* (Chi-square test: *χ*^2^= 2248.2, *df* = 8, *P* < 0.0001) respectively in five boroughs (Bacongo, Makélékélé, Madibou, M’filou and Talangaï), while these values were higher for *Ae. aegypti* compared to *Ae. albopictus* (Chi-square test: *χ*^2^ = 2248.2, *df* = 8, *P* < 0.0001), in four boroughs (Moungali, Ouenzé, Poto-Poto and Djiri) (Fig. 6a, Table 8).

Globally, no significant difference (Wilcoxon rank sum test: *W* = 3, *P* > 0.05) was found between PPI values of *Ae. aegypti* 23.2% (95% CI: 20.4–26.0%) compared to *Ae. albopictus* 19.5% (95% CI: 16.6–22.2%) (Fig. 4a). Similarly, no significant difference (Wilcoxon rank sum test: *W* = 2, *P* > 0.05) was observed between PPI values of *Ae. aegypti* and *Ae. albopictus* in both downtown (47.2%, 95% CI: 41.7–52.7%; 9.8%, 95% CI: 6.7–13%) and suburb (7.9%, 95% CI: 5.5–10.2%; 25.7%, 95% CI: 22.0–29.4%) (Fig. 4b). Nevertheless, analysis according to the boroughs showed that PPI values were higher for *Ae. albopictus* compared to *Ae. aegypti* (Wilcoxon rank sum test: *W* = 0, *P* = 0.002) in Bacongo, Makélékélé and M’filou, while PPI values were higher for *Ae. aegypi* compared to *Ae. albopictus* respectively in Ouenzé, Poto-Poto, (Wilcoxon rank sum test: *W* = 0, *P* = 0.002) and no significant difference was found between both species in Moungali, Madibou, Talangaï and Djiri (Wilcoxon rank sum test: *W* = 5, *P* > 0.05) (Fig. 6b, Table 8).

## Discussion

This detailed study presents a comparative analysis of the ecological adaptation of the two major arbovirus vectors *Ae. aegypti* and *Ae. albopictus*, in Brazzaville, the major city of the Republic of the Congo with a special emphasis on their container preferences, level of infestation, and parameters influencing their distribution and abundance.

Our analysis revealed that both *Ae. aegypti* and *Ae. albopictus* co-exists in all surveyed boroughs in Brazzaville. The co-occurrence of *Ae. aegypti* and *Ae. albopictus* across the Brazzaville city suggests that the environmental factors which prevail in the city are favourable for the development of both species. The presence of *Ae. albopictus* in the Republic of the Congo was confirmed in 2011 during the chikungunya outbreak in Brazzaville [24] suggesting its recent introduction, while *Ae. aegypti* was documented in the country since 1970 [48]. Overall, the prevalence of *Ae. aegypti* in the city of Brazzaville was higher than that of *Ae. albopictus*. This is in accordance with the previous data collected across Brazzaville in May 2017 corresponding to the early rainy season indicating that *Ae. aegypti* was the dominant *Aedes* species in the city [25]. However, the comparative analysis of the prevalence of both *Aedes* species across the city of Brazzaville indicates that *Ae. aegypti* is most prevalent in downtown while *Ae. albopictus* is most prevalent in suburban and rural areas. Indeed, the dominance of *Ae. albopictus* in neighbourhoods located in periurban areas in Brazzaville has been previously reported [38]. These observations are in accordance to previous studies in Central Africa which demonstrated that in sympatric areas, *Ae. aegypti* is most prevalent in neighbourhoods located in downtown with a higher building density, while *Ae. albopictus* is found more frequently in periurban areas surrounded by vegetation [27, 29, 30]. Importantly, seasonality can affect the pattern of abundance of both species as demonstrated previously [27, 49], perhaps due to the difference in the tolerance of desiccation of the eggs of both species [50]. However, previous data collected in Central Africa suggest that this variation depends to the difference of time between the rainy season and the dry season among locations [30].

Larvae of both *Ae. aegypti* and *Ae. albopictus* were found colonizing all container types surveyed across the city. However, peridomestic containers, especially discarded tanks were the most prevalent and the most productive containers for both species. The preference of both species to colonize discarded tanks is in agreement with previous studies in Cameroun [29] and in Central African Republic [27]. These observations are contrary to those generally observed in other regions of the world, particularly in Asia, where *Ae. aegypti* larvae breed commonly in domestic containers such as water storage tanks [44, 51, 52]. Unplanned urbanization and lack of waste management can explain the proliferation of peridomestic containers such as discarded tanks and used tyres in Brazzaville. This can explain why *Ae. aegypti* was found associated with turbid water in this study. The prevalence of water storage containers (27%) observed in Brazzaville suggests a lack of running water in human-dwellings that would promote the storage of water. This prevalence is high compared to that reported in other cities from Central Africa such as Yaoundé and Douala in Cameroon [29].

No significant difference was observed in pupae abundance between the two species according to the container types, except in used tyres for which the abundance of *Ae. albopictus* pupae was higher than the abundance of *Ae. aegypti* pupae suggesting that *Ae. albopictus* infests more used tyres compared to *Ae. aegypti*. Indeed, the invasion of *Ae. albopictus* from Asia to other continents was suggested to be driven by the commercialization of used tyres [22, 53]. Several container-related factors were impacting the presence and or the abundance of *Aedes* immature stages notably the turbidity of water which indicates that the presence of organic matter in the water which can supply food resources. In addition, turbidity of water can also serve to hide aquatic stages of *Aedes* from predators as suggested previously [27, 54]. The presence of the vegetation around the container provides shade which reduces the water temperature in the *Aedes* larval habitat [27, 55]. It was demonstrated that the variation of water volume inside the container, can modulate attractiveness to oviposition, space availability and food resources accessibility [54, 56, 57]. The coexistence of the invasive species *Ae. albopictus* and the native species *Ae. aegypti* in the same ecological niche, implies interspecific competition for resources, which leads to segregation of habitats according to macro-environmental variations such as urban environmental gradients, as shown previously [55, 58] or the decrease of the abundance of the native species [28, 59, 60].

The level of infestation of both vector species, was assessed by calculating *Stegomiya* indices. Traditional *Stegomiya* indices, HI, BI and CI are commonly used to measure the success of vector control strategy to understand the vector ecology. However, these indices are considered by some authors as a poor predictors of epidemiological risk because they are generally not correlated with disease incidence or outbreak [61]. Based on that, Focks & Chadee [62] suggested that pupae based indices such as PI and PPI, which are more epidemiologically relevant indices, could be better predictors for arboviruses transmission; because of their correlation between total pupal densities and adult densities.

Traditional *Stegomyia* indices (BI, CI and HI) and pupal indices (PI, PPI) in our study were relatively high for both species across the city of Brazzaville. Although no significant differences were reported between the index values of the two species at the city scale, all index values significantly varied according to the boroughs and the environment (downtown *vs* suburb). It would be interesting to highlight that this pattern can change according to the season, as mentioned in a previous study in two neighbourhoods of Brazzaville which reported a decrease of *Stegomyia* indices in the dry season compared to the rainy season [38]. The overall BI (38.4%) and HI (26.5%) estimated in Brazzaville for *Ae. aegypti* were higher compare to those estimated in previous studies in other cities from Central Africa such as Yaoundé in Cameroon (BI: 10.1%, HI: 9.0%) [29] and Bangui in the Central African Republic irrespective to the season (early wet season: the BI and HI were 14.4% and 9.03%, respectively; late wet season: the BI and HI were 16.5% and 21.8%, respectively) [27]. Similar observations were reported for *Ae. albopictus* [27]. In addition, index values for both species estimated, were high compared to the reference epidemic thresholds of transmission risk established for yellow fever [42] and for dengue [43]. The high infestation indices of both species suggest a risk for large outbreaks of arbovirus infections such as dengue, yellow fever, Zika, and chikungunya in Brazzaville. Indeed, two large chikungunya outbreaks were reported in the Republic of the Congo during the past decade [13, 34]. During these outbreaks, chikungunya virus was detected in both *Ae. albopictus* and *Ae. aegypti* in 2011 [24], while *Ae. albopictus* was suspected as the main vector during the 2019 outbreak [34]. In addition, it was demonstrated that both *Ae. aegypti* and *Ae. albopictus* from Brazzaville, are able to ensure yellow fever virus [35], dengue virus [37], Zika virus [36] transmission. To clearly establish the epidemiological importance of each *Aedes* species in the Republic of the Congo, additional experiments including feeding behaviour patterns, covering additional locations, and spanning several seasons are needed.

## Conclusions

Our study revealed high infestation rates of *Ae. aegypti* and *Ae. albopictus* across Brazzaville, the major city of the Republic of the Congo, implying a strong potential for human arbovirus infection. Findings generated on the typology, geographical distribution, and productivity of larval development sites of both *Aedes* species could be useful to implement vector control programmes, including management of larval sources by establishing a targeted discarding of most of the productive larval habitats.

## Supplementary information

**Additional file 1: Table S1.** Input files used for modelling binary logistic regression and a negative binomial regression model.

## Data Availability

All data generated or analysed during this study are included in this published article and its additional file.

## Abbreviations

AIC: Akaikeʼs information criterion
BI: Breteau index
CI: container index
GPS: global positioning system
HI: house index
PAHO: Pan American Health Organization
PI: pupae index
PPI: pupae-per-person index
SD: standard deviation
SE: standard error
WHO: World Health Organization
